# Quantitative analysis of the *BRAF* V595E mutation in plasma cell-free DNA from dogs with urothelial carcinoma

**DOI:** 10.1371/journal.pone.0232365

**Published:** 2020-04-24

**Authors:** Michihito Tagawa, Naomi Tambo, Masaki Maezawa, Mizuki Tomihari, Ken-ichi Watanabe, Hisashi Inokuma, Kazuro Miyahara

**Affiliations:** 1 Veterinary Medical Center, Obihiro University of Agriculture and Veterinary Medicine, Obihiro, Hokkaido, Japan; 2 Division of Clinical Veterinary Medicine, Obihiro University of Agriculture and Veterinary Medicine, Obihiro, Hokkaido, Japan; 3 Research Center for Global Agromedicine, Obihiro University of Agriculture and Veterinary Medicine, Obihiro, Hokkaido, Japan; Colorado State University, UNITED STATES

## Abstract

Circulating tumor DNA (ctDNA), which carries tumor-specific mutations, is an emerging candidate biomarker for malignancies and for monitoring disease status in various human tumors. Recently, *BRAF* V595E mutation has been reported in 80% of dogs with urothelial carcinoma. This study investigates the *BRAF* V595E allele concentration in circulating cell-free DNA (cfDNA) and assesses the clinical significance of *BRAF*-mutated ctDNA levels in canines with urothelial carcinoma. A total of 15 dogs with urothelial carcinoma were included. cfDNA concentration was measured using a real-time polymerase chain reaction (PCR) of the *LINE-1* gene. To measure the concentration of the mutated *BRAF* gene in cfDNA, allele-specific real-time PCR with a locked nucleic acid probe was performed. *BRAF* mutations were detected in 11 (73%) of the 15 tested tumor samples. *BRAF*-mutated ctDNA concentrations were significantly higher in dogs with the *BRAF* mutation (14.05 ± 13.51 ng/ml) than in wild-type dogs (0.21 ± 0.41 ng/ml) (*p* = 0.031). The amount of *BRAF*-mutated ctDNA in plasma increased with disease progression and responded to treatment. Our results show that *BRAF*-mutated ctDNA can be detected using allele-specific real-time PCR in plasma samples of canines with urothelial carcinoma with the *BRAF* V595E mutation. This ctDNA analysis may be a potentially useful tool for monitoring the progression of urothelial carcinoma and its response to treatment.

## Introduction

Urothelial carcinoma, also known as transitional cell carcinoma, is the most common form of bladder cancer and an aggressive lower urinary tract tumor in dogs. Reported treatments for urothelial carcinoma include chemotherapy, cyclooxygenase (COX) inhibitors, surgery, and radiation therapy. Urothelial carcinoma is characterized by its invasion of surrounding tissues and its high metastatic potential. Therefore, systemic medical treatment is the mainstay of therapy for urothelial carcinoma in dogs [1]. Although medical therapy is not often curative, remission or stable disease can be accomplished, and median survival times have reached 1 year or greater [2]. Ultrasonography is commonly used for tumor monitoring and measurement in veterinary medicine [1]. However, distension of the urinary bladder can vary over time, making accurate tumor size estimations difficult. Therefore, computed tomography (CT) imaging has been suggested to be more suitable for monitoring of these tumors, especially those with irregular shapes [3]. Unfortunately, this imaging modality can be costly and frequently requires anesthetization.

Circulating cell-free DNAs (cfDNA) are extracellular DNAs that are released into the bloodstream from both normal and tumor cells through apoptosis, necrosis, or direct secretion [4]. Circulating tumor DNAs (ctDNA) released by cancer cells have been found within the cfDNA. These ctDNAs contain the same genetic alterations present in the source tumor and therefore may help clinicians evaluate tumor features in patients with cancer. These ctDNAs may also be useful for monitoring response to therapy through obtaining a simple blood sample known as a liquid biopsy [5]. CtDNAs with *PIK3CA*, *EGFR*, *KRAS*, or *BRAF* mutations have been detected in human patients, and the levels of these ctDNAs have been shown to be useful for the monitoring of disease progression [6, 7].

Recent studies have identified the canine *BRAF* V595E mutation (cBRAF reference sequence ENSCAFT00000006306), which is homologous to the human *BRAF* V600E mutation, in several canine cancers and at least 80% of canine urothelial carcinoma [8, 9]. In humans, approximately 50% of patients with melanoma have the *BRAF* V600E mutation, and *BRAF*-mutated ctDNA levels have been shown to serve as specific biomarkers in these patients [10]. In veterinary medicine, testing for the presence of the *BRAF* mutation using urine or tissue samples is a sensitive and noninvasive method for the diagnosis of canine urothelial carcinoma [11]. However, to our knowledge, no studies have analyzed the use of ctDNA for diagnosis or disease monitoring in canines with urothelial carcinoma. The aim of this study is to measure the *BRAF*-mutated ctDNA levels in plasma and to assess its clinical significance in canines with urothelial carcinoma.

## Materials and methods

### Patients and follow-up

This study was reviewed and approved by the Institutional Animal Care and Use Committee at the Obihiro University of Agriculture and Veterinary Medicine (Permission number: 18–2 and 19–7). Written informed consent was obtained from owners for animal participation in this study. A total of 15 dogs with urothelial carcinoma were included in this study, and all diagnoses were confirmed by histology or cytology at the Veterinary Medical Center at the Obihiro University of Agriculture and Veterinary Medicine (VMC-OUAVM) between February 2018 and May 2019. Staging of the urothelial carcinoma was performed according to the World Health Organization (WHO) staging system [12], which incorporates the use of CT imaging, thoracic radiography with three views, and abdominal ultrasonography. Patients were treated at the discretion of their owners and veterinarians, as appropriate for their disease stage. Tumor responses were assessed at each visit by Response Evaluation Criteria in Solid Tumors (cRECIST) version v1.0 [13], and were classified as having a complete response (CR), partial response (PR), stable disease (SD), or progressive disease (PD).

### Blood collection and cell-free DNA extraction

Two milliliters of peripheral blood in ethylenediaminetetraacetic acid were collected from each dog. Plasma was then separated by centrifugation at 2000 *g* for 10 min at 4 ˚C, transferred to new tubes, and centrifuged at 16,000 *g* for 10 min at 4 ˚C to remove cell debris. Plasma was stored at -30 ˚C prior to DNA extraction. All samples were processed within 4 h of blood collection. cfDNA was isolated from 500 μl of plasma using the MagMAX Cell-Free DNA Isolation Kit (Thermo Fisher Scientific, Inc., Waltham, MA, USA) according to the manufacturer’s instructions. The cfDNA preparations were eluted in 50 μl of elution buffer and stored at -30 ˚C until further analysis.

### BRAF mutation status

The *BRAF* mutation status was available for all dogs and was obtained from DNA from biopsy or surgical specimens using the QIAamp DNA Mini Kit (QIAGEN, Hilden, Germany). To determine the sequence of c*BRAF*, a polymerase chain reaction (PCR) was performed, as previously described [9]. Sequencing was performed at Eurofins Genomics (https://www.eurofinsgenomics.jp/), and data were analyzed for the presence of the c*BRAF* V595E mutation using Sequencher (Sequencher® version 4.7 DNA sequence analysis software, Gene Codes Corporation, Ann Arbor, MI, USA).

### Quantification of cfDNA using the *LINE-1* gene

The plasma cfDNA concentration was measured using a quantitative real-time PCR (qPCR) reaction of long interspersed nuclear element-1 (*LINE-1*), as previously described [14]. The results of this assay were used for the normalization of DNA volume to be used in the qPCR for the *BRAF* mutation. Primer sequences were as follows: forward primer 5’-AAATGCAATGAAACGCCGGG-3’ and reverse primer 5’-TCTTTCGTTGGACACCGAGG-3’ [15]. The qPCR reaction was performed in a 20 μl total volume, containing 500 nM of each primer, 1 μl of cfDNA template, and 10 μl of PowerUp SYBR Green Master Mix (ABI; Thermo Fisher Scientific, Inc., Waltham, MA, USA), using a StepOne Real-Time PCR System (ABI). Initial incubation was performed at 50°C for 2 min and 95°C for 2 min, followed by 40 cycles of denaturation at 95°C for 3 sec and annealing/extension at 60°C for 30 sec. A melt curve (60–95°C) was generated at the end of each run to verify specificity. A standard curve generated by a 10-fold serial dilution (from 1.0–10,000 ng/ml) of genomic DNA obtained from peripheral blood leukocytes of a healthy dog was used to determine the absolute equivalent amount of cfDNA in each sample. All samples were evaluated in duplicate, and a negative control (without template) was included in each plate.

### Allele-specific qPCR to measure percentage of the *BRAF* mutation

**Fig 1:**
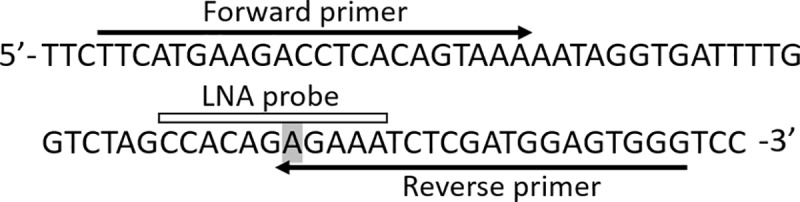
Primer and LNA probe position for amplification of mutated *BRAF* DNA. Gray area indicates the mutated allele.

Primer pairs were designed for the measurement of the *BRAF* mutation in cfDNA. Forward primer (5’- TTCATGAAGACCTCACAGTAAA-3’) was common to wild-type, while the reverse primer (5’-CCCACTCCATCGAGATTTCT-3’) was specific to the mutated sequence. A dual-labeled locked nucleic acid (LNA) probe (5’-FAM-CCA[+C]A[+G][+A][+G]A[+A]A[+T]C-IABkFQ-3’, Integrated DNA Technologies, Coralville, Iowa, USA) for detecting the *BRAF* mutation gene sequence was also designed (Fig 1).

**Fig 2:**
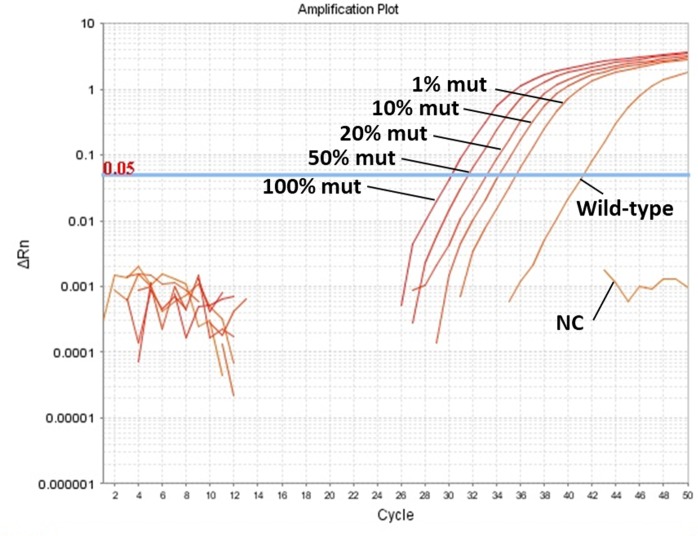
Amplification plots obtained for wild-type and for samples containing known percentages (100%, 50%, 20%, 10%, and 1%) of *BRAF* mutated samples (mut). NC means negative control using distilled water.

To measure the percentage of the *BRAF* mutation in plasma samples, qPCR was performed in duplicate with 0.5 ng plasma cfDNA in a 20 μl total volume, containing 500 nM of each primer, 250 nM of LNA probe, 5 μl of cfDNA template, and 10 μl of PrimeTime Gene Expression Master Mix (Integrated DNA Technologies). Initial incubation was performed at 95°C for 3 min, followed by 50 cycles of denaturation at 95°C for 5 sec and annealing/extension at 63°C for 30 sec. The standard curve for the *BRAF* mutation consisted of six dilutions (100%, 50%, 20%, 10%, 1%, and 0% of *BRAF* mutated samples) obtained by mixing DNA from a mutant transitional cell carcinoma cell line (LC-TCC) and wild-type genomic DNA obtained from peripheral blood leukocytes of a healthy dog (Fig 2). Sample results were expressed as percentages of mutation. Because canine TCC is heterozygous for the *BRAF* mutation, mutated *BRAF* concentrations were calculated as follows: mutated *BRAF* ctDNA (ng/ml) = (% mutated *BRAF* × total cfDNA)/100/2 [16]. Analysis of 3 different runs provided the following values (mean± S.D.): slope = –3.36±0.11 (efficiency = 98.67±4.40), R^2^ = 0.96±0.04, and *Y*-intercept = 38.97±0.94.

### Statistical analyses

Statistical analyses were carried out using JMP 13 software (SAS Institute, Cary, NC). The Mann-Whitney *U* test and Kruskal-Wallis test were applied to compare plasma cfDNA concentrations between groups. The Steel-Dwass test was performed on each pair of groups after the Kruskal-Wallis test. Survival time was defined as the time from study entry to date of death or last follow-up. Dogs alive at the last follow-up were censored for survival analysis. Survival curves were calculated by the Kaplan-Meier method using median values as the cut-off. The generalized Wilcoxon test was used for survival estimates. A *p*-value of less than 0.05 was considered statistically significant.

## Results

### Patient characteristics

This study included 15 patients with 7 males (nonintact) and 8 females (1 intact). Patients had a median age of 12 years (range 8–15 years). Ten dogs were diagnosed with urothelial carcinoma by histology, and five dogs were diagnosed by cytology. *BRAF* mutations were detected in 11 (73%) of the 15 tested tumor tissue or urine samples (S1 Fig). Metastases at sampling time were observed in six dogs (lymph node, n = 3; lung, n = 2; and bone, n = 1). The location of the primary tumor was noted in all patients. Eleven tumors were located purely in the bladder, three were located in the urethra with extension into the bladder, and one was located in the prostate. The clinical characteristics of each patient are summarized in Table 1.

**Table 1:** Characteristics of patients.

Characteristics	Total (n = 15)	V595E (n = 11)	Wild-type (n = 4)
**Sex**			
Female	8	6	2
Male	7	5	2
Age*	12 (range; 8–15)	12 (range; 8–15)	12 (range; 10–13)
**Location**			
Bladder	11	9	2
Bladder+urethra	3	1	2
Prostate	1	1	0
**Tumor stage**^**a**^			
T2	11	8	3
T3	4	3	1
**Lymph node**^**a**^			
N0	12	9	3
N1	3	2	1
**Metastasis**^**a**^			
M0	12	9	3
M1	3	2	1

a) According to the WHO staging system [12]. T2, tumour invading the bladder wall; T3, tumor invading neighboring organs; N0, no evidence of lymph node involvement; N1, lymph node involvement; M0, no evidence of distant metastasis; M1, distant metastasis.

*: years

### CfDNA concentration

**Fig 3:**
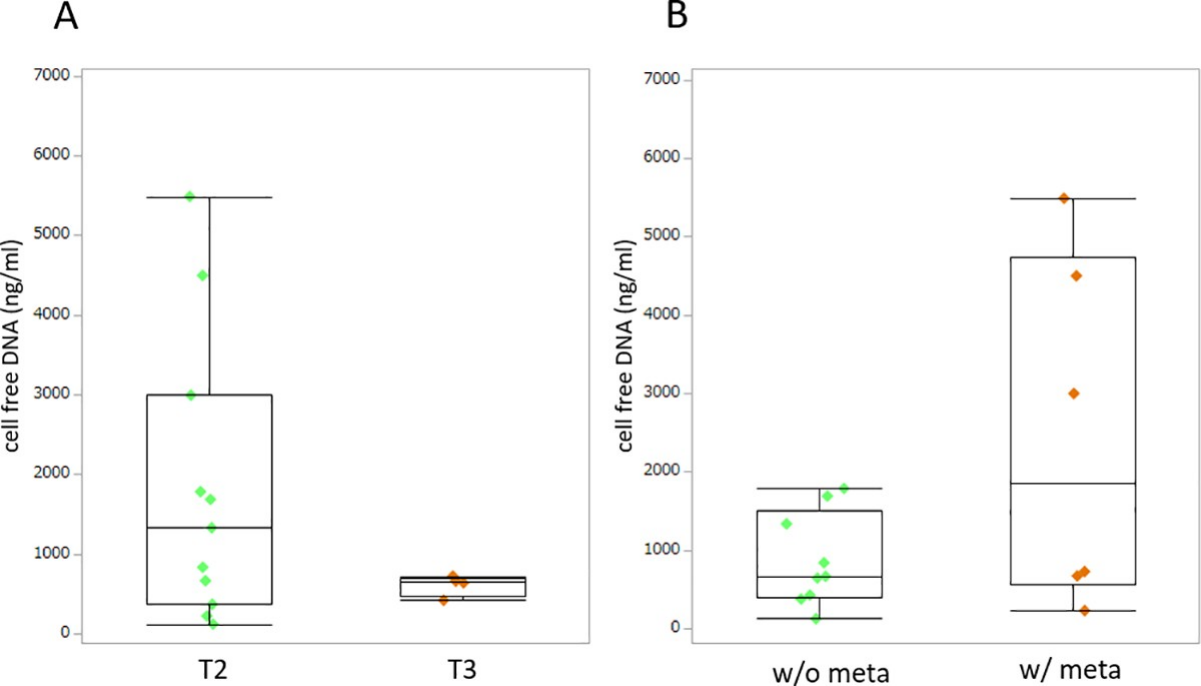
Box-plot of plasma cell-free DNA concentrations in dogs with urothelial carcinoma. Each box indicates the 25th and 75th percentiles. The horizontal line inside the box indicates the median, and the whiskers indicate the extreme measured values. A. Local tumor stage. T2: tumor invading the bladder wall; T3: tumor invading neighboring organs. B. Metastasis condition. w/o meta: no evidence of metastasis; w/ meta: metastasis present.

The median cfDNA concentration of *LINE-1* in all dogs at presentation was 724.3 ng/ml (range 122.2–5487.6 ng/ml). We compared cfDNA concentrations of dogs with different tumor stages and found no significant difference between T2 stage (n = 11; mean ± SE: 1819.07 ± 1790.95 ng/ml) and T3 stage (n = 4; 612.63 ± 131.32 ng/ml) (*p* = 0.240) (Fig 3). Plasma cfDNA concentration was higher in dogs with metastasis (2433.05 ± 2227.92 ng/ml) than in dogs without metastasis (873.55 ± 594.00 ng/ml); however, no significant difference was found (*p* = 0.195) (Fig 3).

### Mutated *BRAF* percentage and concentrations

**Fig 4:**
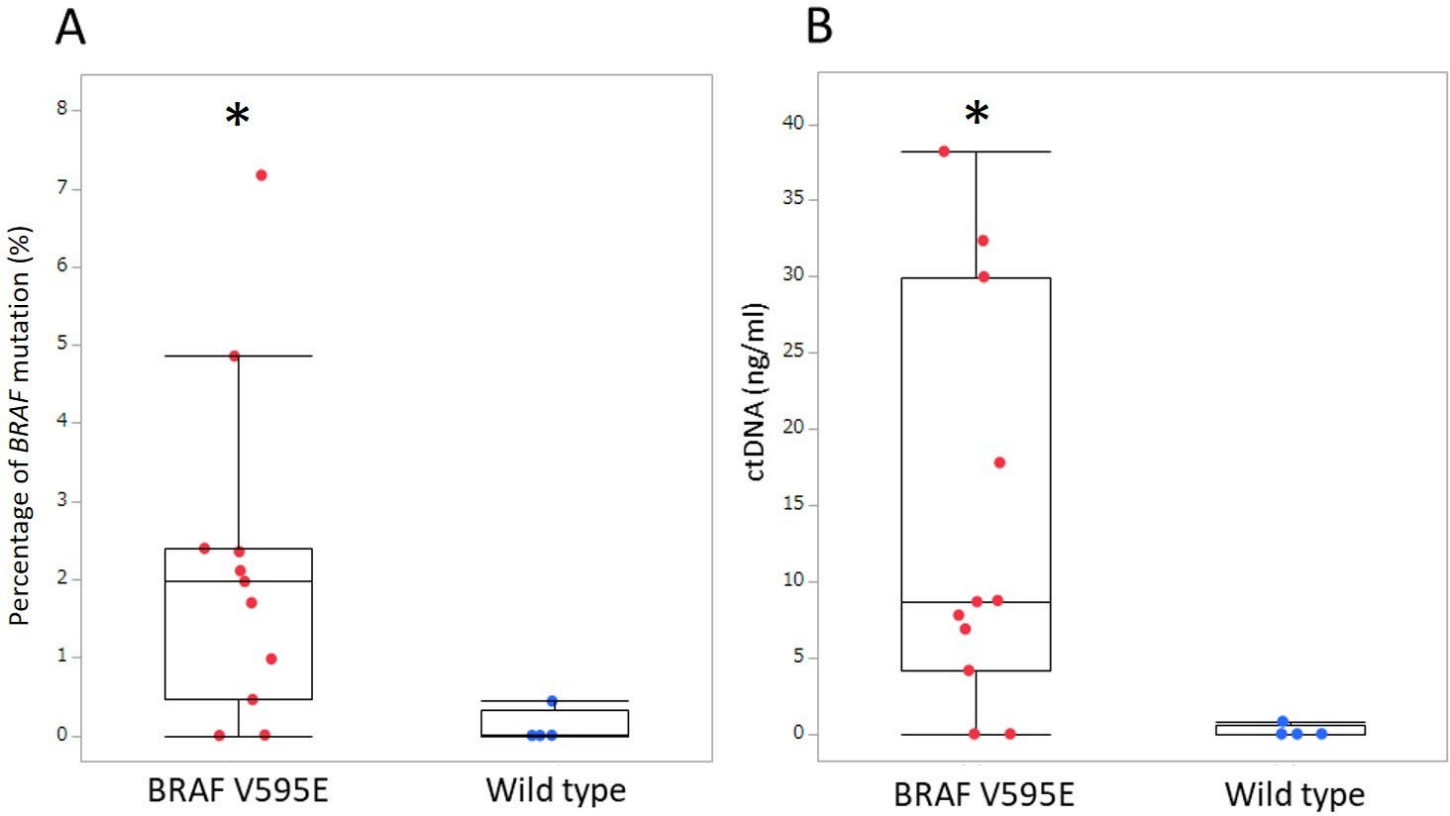
Box-plot of (A) percentage of the *BRAF* mutation and (B) *BRAF*-mutated ctDNA concentration in plasma obtained from dogs with urothelial carcinoma. Each box indicates the 25th and 75th percentiles. The horizontal line inside the box indicates the median, and the whiskers indicate the extreme measured values. * *p* < 0.05.

In order to evaluate the sensitivity of the qPCR assay, we performed 3 replicates using the standard in the same assay run. The theoretical detection limit of the assay was determined to be 0.13% *BRAF*-mutated ctDNA. The mean percentage of the *BRAF* mutation in cfDNA was 2.18 ± 2.15% in dogs with the *BRAF* mutation and 0.11 ± 0.22% in wild-type dogs (*p* = 0.022). *BRAF*-mutated ctDNA concentration was significantly higher in dogs with the *BRAF* mutation (14.05 ± 13.51 ng/ml) than in wild-type dogs (0.21 ± 0.41 ng/ml) (*p* = 0.031) (Fig 4).

**Fig 5:**
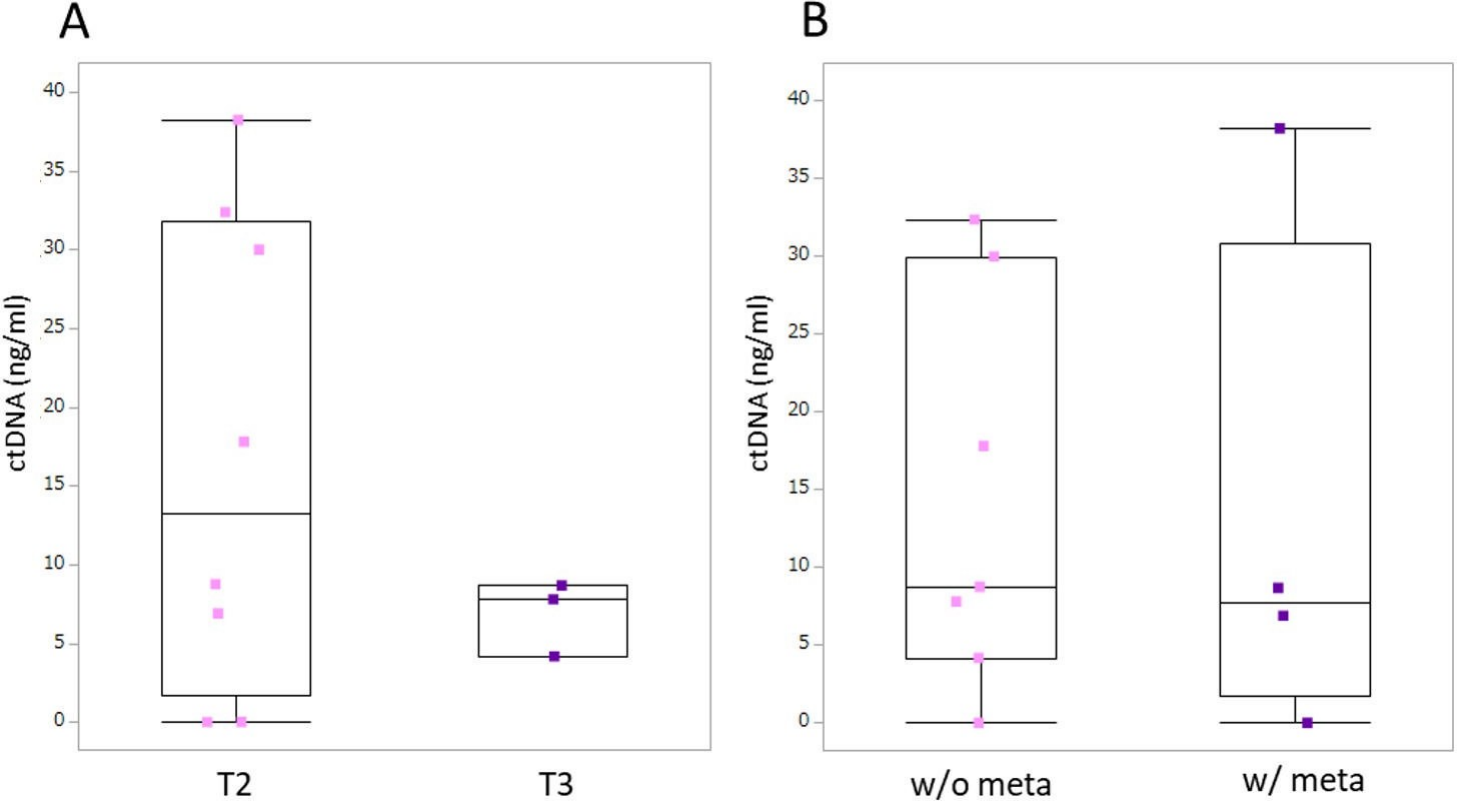
Box-plot of *BRAF*-mutated ctDNA concentrations in plasma obtained from dogs with urothelial carcinoma. Each box indicates the 25th and 75th percentiles. The horizontal line inside the box indicates the median, and the whiskers indicate the extreme measured values. A. Local tumor stage. T2: tumor invading the bladder wall; T3: tumor invading neighboring organs. B. Metastasis condition. w/o meta: no evidence of metastasis; w/ meta: metastasis present.

We compared the *BRAF*-mutated ctDNA concentrations in 11 dogs with the *BRAF* mutation with different tumor stages. No significant difference was found in *BRAF-*mutated ctDNA concentrations identified between dogs with T2 (n = 8; 16.75 ± 15.13 ng/ml) or T3 (n = 3; 6.88 ± 2.39 ng/ml) (*p* = 0.475) disease. Dogs with metastasis (n = 4) also had no significant difference in their *BRAF*-mutated ctDNA concentration (13.44 ± 16.92 ng/ml) compared to dogs without metastasis (n = 7; 14.41 ± 12.68 ng/ml) (*p* = 0.777) (Fig 5).

### Plasma DNA dynamics and tumor progression

The levels of *BRAF*-mutated ctDNA were assessed in six patients during the treatment course (Table 2). In several patients, the concentration of *BRAF*-mutated ctDNA in plasma increased with disease progression and responded to the treatment.

**Table 2:** Clinical characteristics of six patients assessed by follow-up study.

Patient	Breed	Age	Gender	Tumor site	Stage^a^	Metastasis site	Treatment	ST (days)
1	MP	9	Spayed	Bladder	T2N0M0	No	Surgery+cox2i	22
2	PM	13	Spayed	Bladder	T2N0M0	No	Surgery+cox2i	228*
3	PM	8	Spayed	Bladder	T2N0M0	No	Chemotherapy+cox2i	305
4	Pug	11	Spayed	Bladder+ urethra	T3N0M1	Pelvic bone	Chemotherapy+cox2i	217
5	MD	13	Spayed	Bladder	T3N0M0	No	Chemotherapy+cox2i	340*
6	SS	12	Castrated	Bladder	T2N0M1	Lung	Chemotherapy+cox2i	335*

a) According to the WHO staging system [12].

*: censored case

MP, miniature pinscher; PM, pomeranian; MD, miniature dachshund; SS, shetland sheepdog; ST, survival time; cox2i, cyclooxygenase2 inhibitor

**Fig 6:**
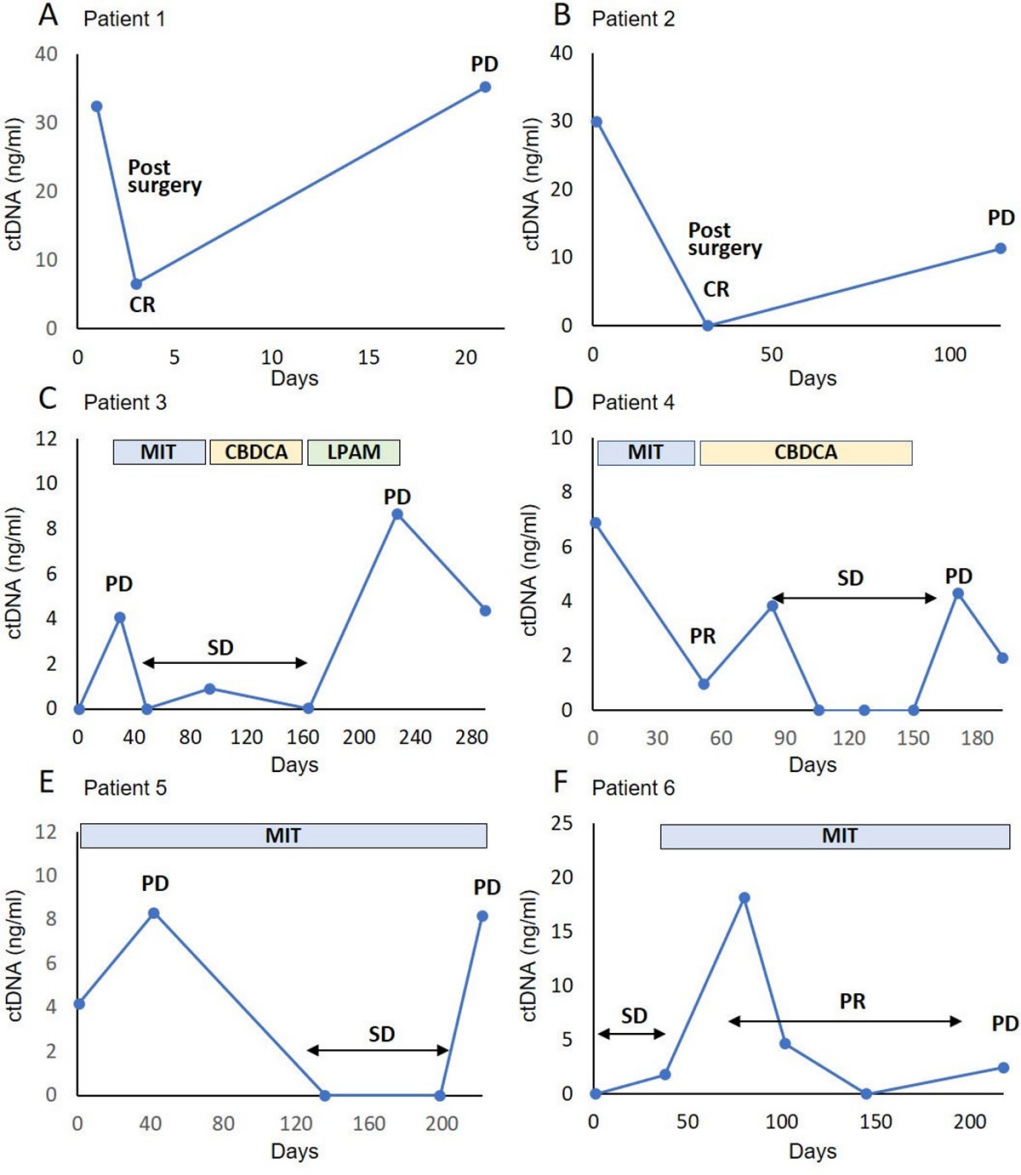
Monitoring of *BRAF-*mutated ctDNA concentrations according to treatment response. Mutated *BRAF* ctDNA levels at various time points in six patients. The vertical axis represents mutated *BRAF* ctDNA concentration, and the horizontal axis represents the time points of plasma-sampling days. All patients were administrated oral cyclooxygenase-2 inhibitors during treatment. CR, complete response; SD, stable disease; PD, progressive disease; PR, partial response; MIT, mitoxantrone; CBDCA, carboplatin; LPAM, Melphalan.

Patients 1 and 2 underwent partial cystectomy after first blood sampling. *BRAF*-mutated ctDNA levels decreased after resection on postoperative day 3 and 32, respectively. Patient 1 died of pneumonia on day 22, and local recurrence was confirmed by necropsy (Fig 6A). In patient 2, bladder recurrence was observed on day 114, and an increase in the mutated ctDNA level was identified (Fig 6B). Both patients were administrated piroxicam, an oral cyclooxygenase-2 inhibitor, after resection.

The owners of patients 3, 4, 5, and 6 chose medical therapy. Patient 3 presented with a lung metastasis on day 30, and an increased *BRAF*-mutated ctDNA level was observed. Progressive primary tumor and lung metastases were observed on day 227 with a substantial increase in the ctDNA levels. The patient’s condition gradually deteriorated, and she died on day 305 (Fig 6C).

Patient 4 presented with a pelvic bone metastasis, and a high level of *BRAF*-mutated ctDNA was detected at first sampling. Reduction of the primary tumor was observed after carboplatin administration, and the ctDNA level significantly decreased on day 52. Skin metastasis appeared on day 150, and rapid tumor progression was seen after day 171. The ctDNA level increased again, and the patient died on day 217 (Fig 6D).

Patient 5 developed PD of her primary tumor, and *BRAF*-mutated ctDNA was detected on day 42. The primary tumor stabilized between days 42 and 199 with mitoxantrone administration, and the DNA level was undetectable. However, tumor progression and bilateral hydronephrosis were observed on day 222, and the ctDNA level increased (Fig 6E).

Patient 6 presented with lung metastases detected on CT scan. Although tumor response was classified as PR on day 80, the *BRAF*-mutated ctDNA level increased between days 80 and 102. Tumor regrowth was detected on day 218, and the ctDNA level increased (Fig 6F).

### Mutated *BRAF* concentrations and survival

The 11 patients who tested positive for the *BRAF* mutation in their primary tumor were divided into two groups according to their plasma concentrations of *BRAF*-mutated ctDNA (< 8.3 ng/ml vs. ≥ 8.3 ng/ml) at first sampling. These thresholds were selected based on the median value of *BRAF*-mutated ctDNA.

**Fig 7:**
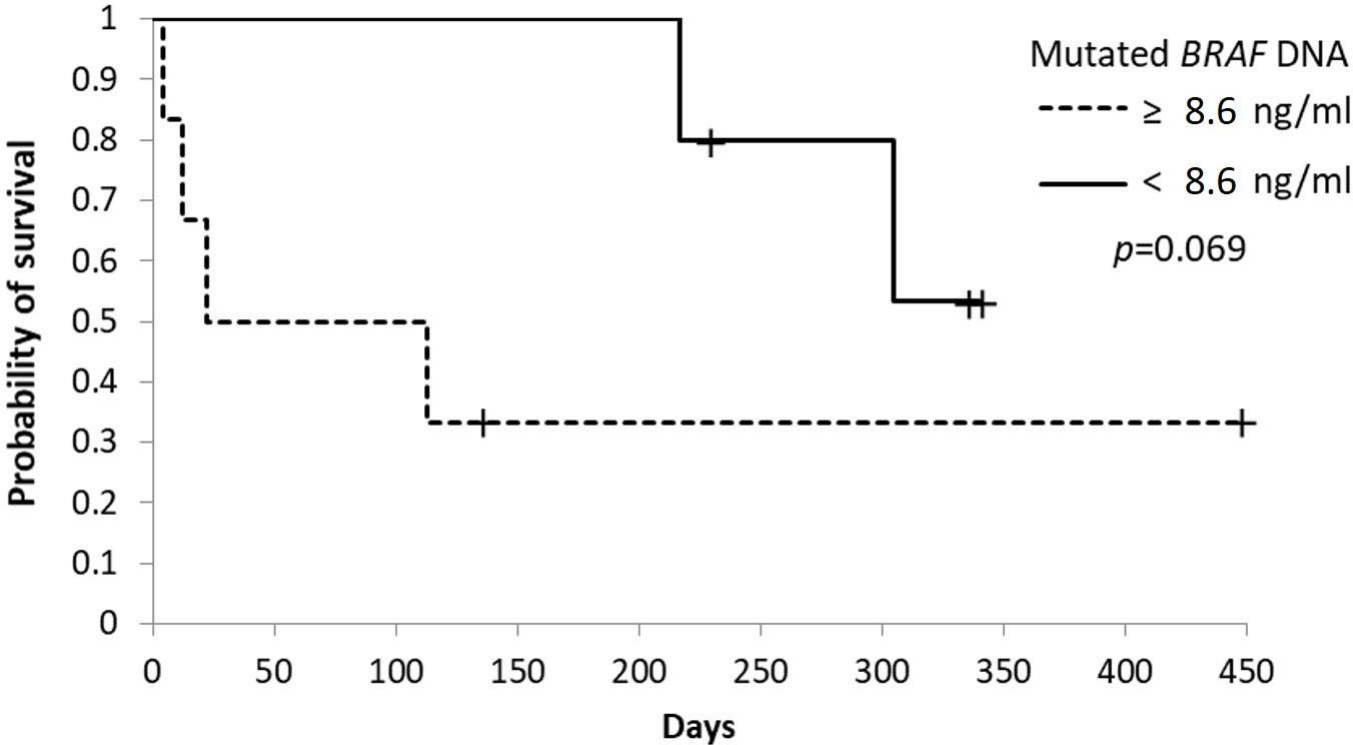
Kaplan-Meier curves of survival time in dogs with urothelial carcinoma according to the cut-off value based on mutated *BRAF* ctDNA concentrations. Cut-off *and p*-values are shown. + censored case.

Although no significant difference was found, the median overall survival duration of the five patients with a *BRAF*-mutated ctDNA concentration of < 8.6 ng/ml was longer than that of the six patients with a *BRAF*-mutated ctDNA concentration of ≥ 8.6 ng/ml (305 vs. 67.5 days, *p* = 0.069) (Fig 7).

## Discussion

CtDNAs carrying tumor-specific mutations have been found in the bloodstream. In humans, ctDNA levels in the plasma or serum are potentially useful noninvasive biomarkers for several tumor types [5]. Recently, studies have reported that the canine *BRAF* V595E mutation, a single nucleotide T to A transversion at nucleotide 1349, is detectable in 80% of canines with urothelial carcinomas [9, 11]. No previous studies have evaluated the measurement of ctDNA levels of this mutation in the plasma of canines with these tumors. In this study, we developed a real-time PCR method using a forward primer common to both wild-type and mutated *BRAF* sequences and a mutation-specific reverse primer. A dual-labelled LNA probe was designed on the strand complementary to that recognized and hybridized by the *BRAF* V595E allele-specific primer. We found that the ctDNA concentration in patients with *BRAF*-mutated urothelial carcinoma is increased compared to that in wild-type patients, demonstrating that the *BRAF* mutation in urothelial carcinoma may be detectable in the plasma of these canines. CtDNAs with mutations in several genes have been detected in human patients with cancer, and ctDNA analysis provides critical clinical information to improve diagnosis in these patients [6, 7, 17].

In this study, the *BRAF* mutation was detected in 11 of the 15 tested tissue or urine samples. Mutated *BRAF* ctDNA was detected in 9 of these 11 patients’ plasma at the time of the first blood collection. *BRAF-*mutated ctDNA was detected in the three other dogs later in the course of their diseases. This study suggests that the quantification of mutated *BRAF* ctDNA in plasma may represent a useful biomarker for the noninvasive diagnosis of canine urothelial carcinoma when the tumor has the *BRAF* mutation. This study also shows that the concentration of mutated *BRAF* ctDNA does not correlate with the clinical stage or the presence of metastases. Past studies have found that ctDNA levels of mutated target genes are higher in patients with more advanced-stage disease [18, 19]. Further studies with larger sample sizes will be necessary to explore this discrepancy. Interestingly, mutated *BRAF* ctDNA was also detected above the cut-off value in one wild-type patient (mutated *BRAF* percentage, 0.44%). In this study, determination of *BRAF* mutant cases was made by Sanger sequencing. Some of cases classified as wild type may have had *BRAF* mutations present at a level below the limit of detection for Sanger sequencing. Furthermore, it is possible that ctDNA in plasma of the dog could be indicative of a *BRAF* mutation originating from other neoplasms that have been undiagnosed because *BRAF* V595E mutation has been detected in not only urothelial carcinoma but also many other neoplasms [9]. In addition, recently, study has reported that low levels of common cancer gene mutations may be present even in healthy individuals [20]. Further clinical validation is essential, however, before being incorporated into clinical practice.

In this study, we observed that mutated *BRAF* ctDNA levels in plasma are approximately correlated with the response to treatment, suggesting that the *BRAF-*mutated ctDNA level may serve as a useful therapeutic biomarker. In humans, previous studies have indicated that mutated ctDNA levels correlate with tumor burden in several tumor types and that mutated ctDNA levels are consistent with the patient’s overall clinical condition [20, 21]. In addition, detection of ctDNA following chemoradiotherapy is associated with tumor progression and postoperative ctDNA detection is associated with recurrence [22, 23]. Detection of mutated *BRAF* cfDNA in plasma may be a potentially useful biomarker for noninvasive follow-up of canines with urothelial carcinoma. In contrast, we also found that mutated ctDNA levels do not reflect the observed disease progression in some patients. From our analysis, whether ctDNA quantification provides useful information for tumor monitoring is still unknown because of our small number of included patients.

Although no significant difference was found, we show that low levels of baseline mutated *BRAF* ctDNA (< 8.6 ng/ml) are predictive of prolonged survival time compared to patients with a high baseline level of mutated *BRAF* ctDNA. Previous reports have showed high or positive baseline levels of ctDNA in various human cancers, which are associated with shorter overall survival and progression-free survival [24, 25, 26]. Since evaluation of ctDNA levels as a prognostic biomarker for canines with urothelial carcinoma has not been reported previously, larger cohort studies are needed to validate our findings.

Since mutated ctDNA derived from malignant tumors exists only at low concentrations in patients, highly sensitive assays are required for assessment. Most PCR methods that detect *BRAF* mutation are limited by the presence of a large proportion of wild-type *BRAF*, which interferes with ctDNA mutation assays [27]. Currently, digital PCR and next-generation sequencing are the main methods used to detect mutation abundance in cfDNA due to their high sensitivities and specificities [20]. However, both methods require specialized instruments and high costs to perform multiple tests for regular tumor monitoring. For this study, we developed an allele-specific real-time PCR method with a dual-labelled LNA probe. The method has already been used to detect *BRAF* mutations in plasma samples of human patients with melanoma [16]. Although we included only a small number of patients, changes in plasma mutated *BRAF* ctDNA levels associated with tumor progression were successfully detected by our method. Further studies are needed to assess the clinical value of ctDNA quantification using this method in dogs with urothelial carcinoma.

A limitation of the present study was the small number of patients. The small sample size used for each analysis may have limited the extent to which differences between groups could be detected. Additionally, our study was a retrospective analysis, and therapeutic methods were not consistent. Therefore, a future large-scale prospective study using standardized treatment modalities will be necessary for adequate evaluation.

In conclusion, our results show that mutated *BRAF* ctDNA can be detected using allele-specific real-time PCR in plasma samples of canines with urothelial carcinoma harboring the *BRAF* V595E mutation. This technique may be useful for monitoring disease progression and treatment response. Further research is required to establish the true clinical value of plasma mutated *BRAF* ctDNA as a biomarker for canine urothelial carcinoma.

## Supporting information

S1 Fig Sequence analyses of the *BRAF* gene from dogs with urothelial carcinoma. (TIFF)
