# Supplementary figures and images for: Quantitative analysis of the BRAF V595E mutation in plasma cell-free DNA from dogs with urothelial carcinoma
Source: PLoS One. 2020 Apr 24;15(4):e0232365. doi: 10.1371/journal.pone.0232365 (PMC7182225; doi:10.1371/journal.pone.0232365)

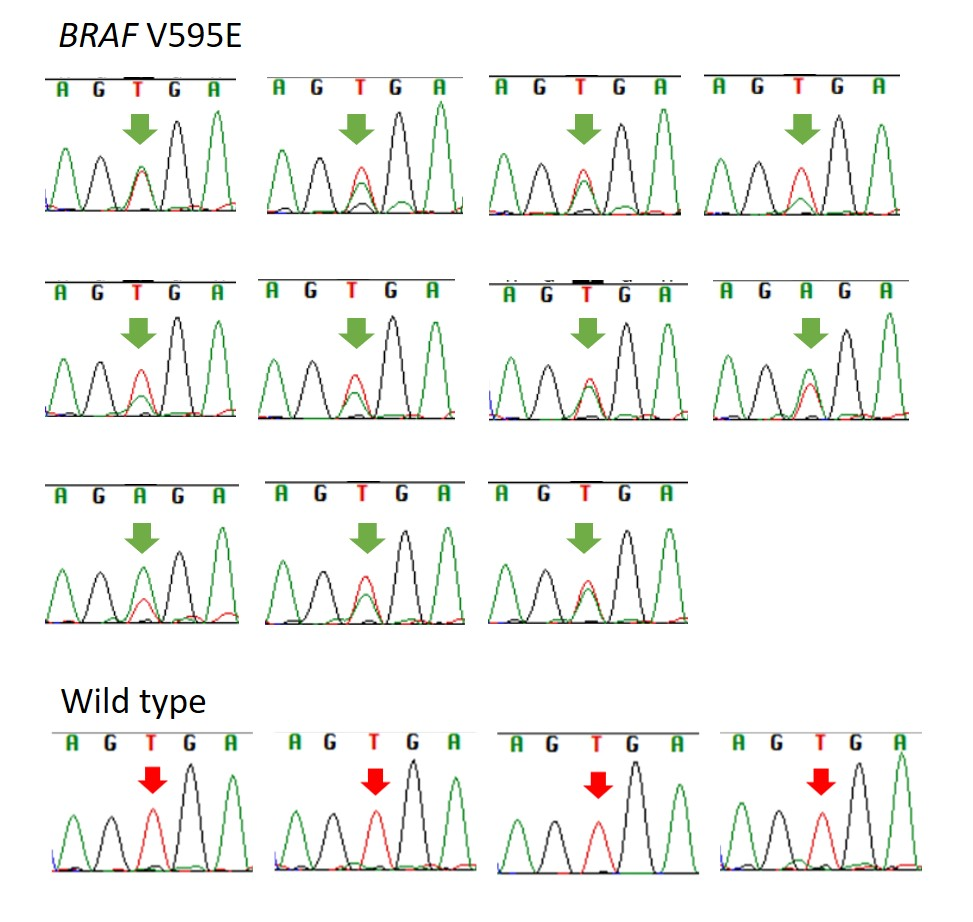

Supplement: S1 Fig — (TIFF) [file pone.0232365.s001.tiff]
